# Double-application of platelet-rich plasma on bone healing in rabbits

**DOI:** 10.4317/medoral.17336

**Published:** 2011-12-06

**Authors:** Burcu Özdemir, Bülent Kurtiş, Gülay Tüter, Burcu Sengüven, Benay Tokman, Selin Pınar-Özdemir, İlkim Demirel, Gönen Özcan

**Affiliations:** 1 Gazi University Faculty of Dentistry, Department of Periodontology, Ankara, Turkey; 2 Gazi University Faculty of Dentistry, Department of Pathology, Ankara, Turkey

## Abstract

Objective: Platelet-rich plasma (PRP) is considered to enhance bone formation especially at early stages of wound healing, depending on the limited and short life-span of platelets and growth factors. The aim of this study was to evaluate efficacy of double-application of PRP (DA-PRP) on bone healing in a rabbit calvarial defect model. Study design: Twenty-eight rabbits, each had two surgically prepared calvarial bone defects (10mm diameter), were included in this study and randomly divided into six groups. Defects (n=56) were treated with single-application of PRP (SA-PRP)(n=10), SA-PRP and beta-tricalciumphosphate (SA-PRP+TCP)(n=10), DA-PRP (n=8), DA-PRP and beta-tricalciumphosphate (DA-PRP+TCP)(n=8), beta-tricalciumphosphate (TCP)(n=10) or left empty (Control)(n=10). Animals were sacrificed at 30 days postoperatively. Results: The new bone (NB%) and defect fill (DF%) percentages were calculated from histological slides by image-analyzer software and statistically analysed. All test groups showed higher NB% than control, but differences among all groups were insignificant. The TCP treated groups had significantly higher DF% than groups treated without TCP, however the DF% differences between control, SA-PRP and DA-PRP or TCP, SA-PRP+TCP or DA-PRP+TCP were insignificant. Conclusion: Although new bone formation was histomorphologically remarkable at double-application PRP groups, statistical analyses of the histomorphometric data revealed no significant difference.

** Key words:** Platelet-Rich Plasma, double application, bone formation, wound healing.

## Introduction

Lately, platelet rich plasma (PRP), an autologous platelet concentration, suggested to have a potential to increase regeneration and accelerate wound healing, due to the various growth factors it consists (1-4). PRP application, previously, has been showed to increase the local platelet concentration by 338% (5) and consequently increase the local growth factor concentration(6). Platelet derived growth factor, transforming growth factor-beta, insulin like growth factor, endothelial growth factor, fibroblast growth factor and vascular endothelial growth factor are some of the growth factors described in PRP (5,7,8). Combination of two or more growth factors are already known to be more effective on many cell types compared to single growth factors (9). There are numerous encouraging reports in the favour of PRP (1,10,11), however PRP is still a terra incognita in many terms. 

It remains a question that how long PRP is effective. Regarding short life-span of platelets and platelet derived growth factors, PRP is believed to be effective predominantly at the early stages of the hard tissue healing.(5,12) Degradation of platelets and growth factor release reported to be at first 3-5 days and therefore growth factor activity is suggested to be at first 7-10 days.(12) It was also suggested that direct effects of platelet derived growth factors start to disappear gradually after 5-6 days.(5) Also, it is still unknown that how many times PRP should be applied to the wound area or do multiple applications improve the results. 

Crovetti et al. (2004) treated 24 cutaneous chronic nonresponsive severe ulcers with multi-application of PRP topically and reported 9 to be healed completely and 8 healed >50% (13). Driver et al. (2006) compared application of PRP gel with saline gel (control) topical dressing in themulti treatment of nonhealing diabetic foot ulcers (14). They reported that treated wounds healed significantly more in PRP gel group (13 out of 16, 81.3%) than in control group (eight out of 19, 42.1%) (P = 0.036) (14). Radaelli et al. evaluated the outcomes, benefits and side effects of a standardized PRP injection protocol in a series of 23 consecutively treated patients and reported that results were promising for face and neck revitalization (15). More recently Kon et al. (2010) evaluated efficacy of PRP at the treatment of degenerative lesions of articular cartilage of knee after multi intra-articular PRP injections and their preliminary results indicated that treatment with PRP injections was safe and has the potential to reduce pain and improve knee function (16). 

Considering the limitations of autogenous bone and allografts, such as limited availability of donor sites and enhanced risk of disease transmission, respectively, alloplastic grafts appears to be safe and convenient (17-21). Beta-tricalcium phosphate (b-TCP), which has interconnected system of micropores, Ca/PO4 ratio similar to a natural bone and approximately 12 months of resorption time in human intrabony defects, has been widely used as a biologically safe osteoconductive alloplastic bone substitute (1,22-24). The clinical and experimental studies examining the effects of PRP and b-TCP together reported that both b-TCP alone and PRP/b-TCP were successful at the treatment of intrabony defects, howe-ver those studies had conflictive conclusions about additional effects of PRP: Some of the authors reported that PRP had significantly improved the results achieved by b-TCP alone (10,11,25), whereas others claimed that PRP had no additional benefits to b-TCP treatment at intrabony defects (19,26). 

Although numerous studies evaluated single application of PRP and/or graft-PRP combinations at the treatment of intrabony defects (3,7,10,27), there is still lack of information on efficacy of multi-application of PRP on osseous defects. Within the limitations of our know-ledge, the present study appears to be the first study to examine double-application of PRP alone and together with b-TCP in the treatment of bone defects. 

The purpose of the present study was to evaluate efficacy of double-application of PRP (DA-PRP) on bone healing in a rabbit cranial defect model, alone and in combination with b-TCP, and to compare it with single-application of PRP and empty controls by histological methods.

## Material and Methods

Twenty-eight healthy 6-months-old female New Zealand rabbits weighing between 3-4 kg were used as experimental animals for the study. Before the study, general health of the rabbits was monitored for 10 days. The rabbits were kept in standard cages in an experimental animal room and were fed a standard laboratory diet and water. This study protocol was approved by the ethical committee for animal experiments of the Gazi University, Ankara, Turkey (G.Ü.ET-06.043).

 -PRP Preparation 

Sterile disposable monovette system (Curasan, Pharma Gmbh AG, Lindigstrab, Germany ) and compatible centrifuge machine (Heraeus Labofuge 300, Kendro Laboratory Products, D-37520 Osterrade, German ) was used for preparations of PRP. 

Eight ml of peripheral blood was drawn from each animal by venipuncture and transferred into red marked monovette containing 0.5 ml citrate (10% trisodium citrate), approximately 30 min before the surgery. Monovette is centrifuged at 2400 rpm for 10 min. After the first centrifugation, two layers were seen clearly in the monovette. Previously it was reported that upper yellow layer was consist of platelet rich and poor plasma and lower red layer was consist of erythrocytes and leukocytes.(2) And also lower red layer’s top 1-2 mm part was reported to be rich from platelets which newly joined to the circulation.(3,4) A total of 4 mm plasma, which consisted of complete upper yellow layer and also lower red layer’s top 1-2 mm part, transferred into a yellow marked monovette. After the second centrifugation at 3600 rpm for 15 min, approximately 0.7 mm was plasma rich from platelets at bottom of the monovette and the upper rest was plasma poor from platelet.(2) Part of the platelet poor plasma was collected and discarded. Remaining 0.7 ml plasma at the bottom of the yellow marked monovette vortexed for 20 sec and transferred into green marked application injector. This final preparation of PRP (2,28) was ready to use. 

 -Surgical Procedure

All surgical procedures were performed under aseptic conditions in an animal-operating suite at Gazi University. The rabbits were anaesthetized with an intramuscular dose of 35mg/kg ketamine (Ketanes, Alke, İstanbul, Turkey) and 5mg/kg xylazine (Rompun, Bayer, Leverkusen, Germany). Animals were placed in sterna recumbency. Their head was shaved and the cutaneous surface was disinfected with a povidone iodine solution prior to surgery. The calvaria bone was exposed after incision of skin and periosteum, respectively. Two circular calvarial bone defects (0,5mm thick x 10mm inner diameter) were made in parietal bone, on each side of the median sagittal suture without crossing it, using a trephine bar on a slow-speed electric hand piece by applying physiologic saline irrigation without injuring the underlying duramater.

**Figure 1:**
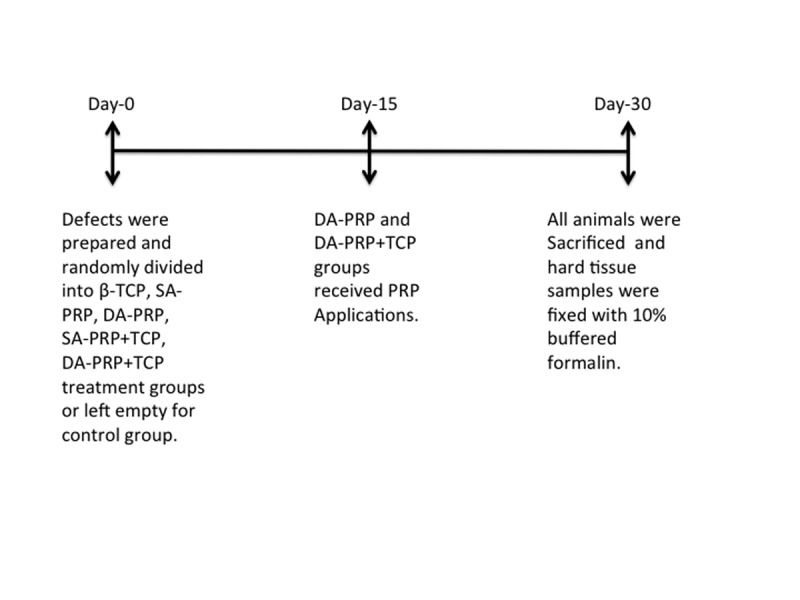
Study design.

The rabbits were randomly divided into six groups and cranial defects (n=56) were treated with single-application of PRP (SA-PRP) (n=10), SA-PRP and beta-tricalciumphosphate (β-TCP) graft (SA-PRP+TCP) (n=10), DA-PRP (n=8), DA-PRP and β-TCP (DA-PRP+TCP) (n=8), β-TCP (TCP) (n=10) or left empty (control) (n=10). Study design was summarized at (Fig. 1).

PRP was prepared before the operations, as described above, at all PRP groups on Day-0 of the study. Regarding PRP applications for SA-PRP and DA-PRP groups, final PRP preparation was mixed with autogenous blood taken from defect area for activation of platelets, approximately 5 min prior to application to the defect area. As for preparation of PRP+TCP combinations for SA-PRP+TCP and DA-PRP+TCP groups, β-TCP (CerasorbÒ, pure β-TCP granules, 0.5gr, granule size 500-1000µm, Curasan, Kleinostheim, Germany) was transferred into sterile glass godet then blood taken from defect site and PRP were added and mixed. PRP/β-TCP combination was ready for application after approximately 5 min. The skin was relocated with silk continuous sutures (3/0) following the closure of periosteum with resorbable (4/0) sutures (Pegesorb, Doğsan, Trabzon, Turkey ). Post-operative analgesics were administered as intramuscular ketoprofen (Profenid, Eczacıbaşı, İstanbul, Turkey ) 3mg/kg 1x1 for 3 days. 

Second PRP preparation for DA-PRP and DA-PRP+TCP groups took place at Day-15, which was transferred to the same defect areas via injection. The animals were sacrificed 4 weeks after the initial intervention with an intravenous overdose of a combination of ketamine and xylazine. The calvarium bone including defect and surrounding bone was dissected and placed into the 10% formalin buffer solution at pH 7.0 before further analysis. Samples transferred to the laboratory for histological examination.

 -Histological evaluation 

All histological procedures and evaluations were carried out at the Department of Oral Pathology, Faculty of Dentistry, Gazi University. Hard tissue samples were fixed with 10% buffered formalin for 24–72 hours and decalcified with 10% formic acid for 3 weeks. After washing under tap water overnight, samples were embedded in paraffin. Three sections with 4µm thickness were cut at the central region of each specimen to obtain maximum standardization of the cutting surface. All sections were deparaffinised at 56oC and by xylene then incubated in absolute and 96% ethanol. For histomorphological evaluation, sections were stained with routine hematoxylene-eosine (H&E). 

All histological evaluations of stained sections were carried out using Leica DM 4000 B microscope (Leica Microsystems GmbH. Wetzlar, Germany) and Leica QWin Plus v3.3.1 image analyzer programme (Leica Microsystems GmbH. Wetzlar, Germany). The following measurements were taken from each histological central section: dimensions of total defect area, newly formed bone and remaining graft material areas. These data permit to calculate defect fill (DF %) and new bone percentages (NB %):

New Bone (NB%) = New bone area / Total defect area x 100

Defect Fill (DF%) = New bone area + remaining area of graft materials / Total defect area x 100

 -Statistical analysis

Statistical analysis was performed by Statistical Package for Social Sciences (SPSS) 11.5 software (SPSS Inc., Chicago, IL, United States ). Whether the continuous variables were normally distributed or not was determined by using Shapiro Wilk test. Data were expressed as median and (25th – 75th) percentiles. Differences among six groups regarding for continuous date were evaluated by Kruskal-Wallis variance analysis. When the p-value from the Kruskal-Wallis test statistics is statistically significant, Conover’s multiple comparison test was used to know which groups differ from which others.(29) A p value less than 0.05 was considered as statistically significant.

## Results

In all groups, different percentage of connective tissue, new bone formation, and graft particles were seen in defected areas. All groups except for control sections showed bone formation throughout the bone defect. 

**Figure 2:**
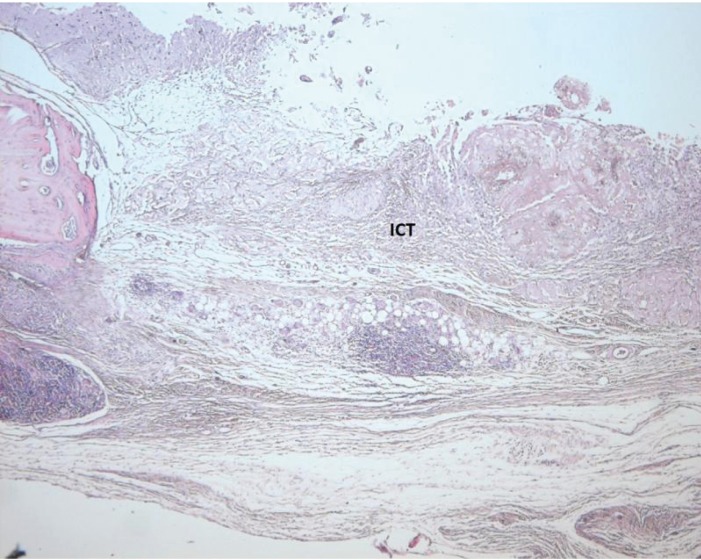
Light microscope photograph of a section from. Control group. The inflamed connective tissues (ICT) are seen (H&E × 100).

**Figure 3:**
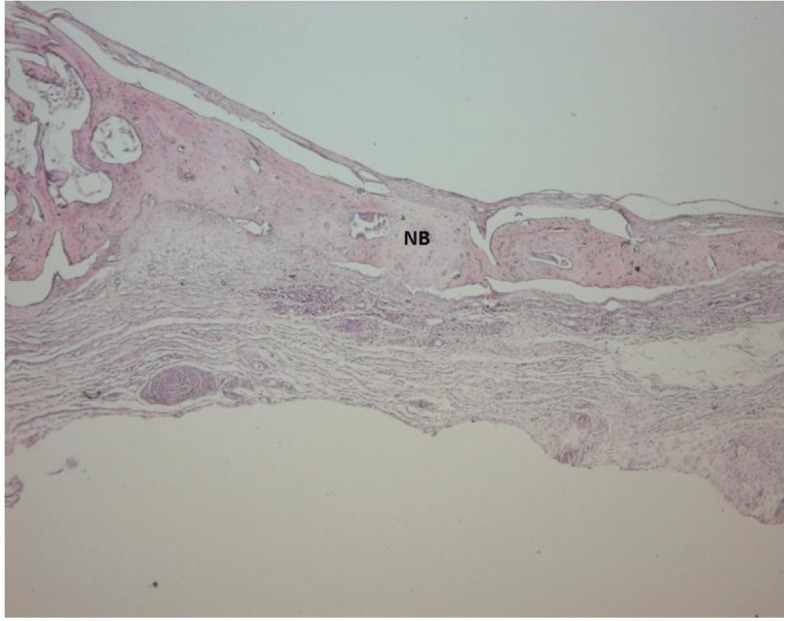
Light microscope photograph of a section from SA-PRP group. The cellular new bone (NB) are seen (H&E × 100).

**Figure 4:**
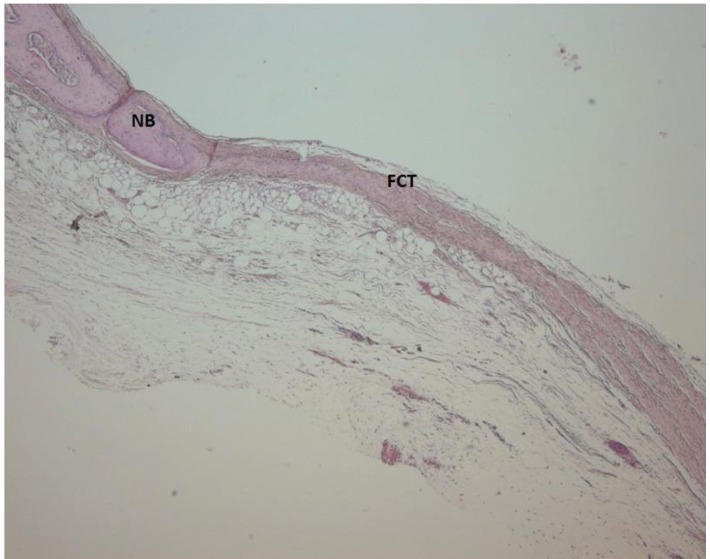
Light microscope photograph of a section from DA-PRP group. The new bone (NB) can be seen over the fibrous connective tissue (FCT) ground (H&E × 100).

**Figure 5:**
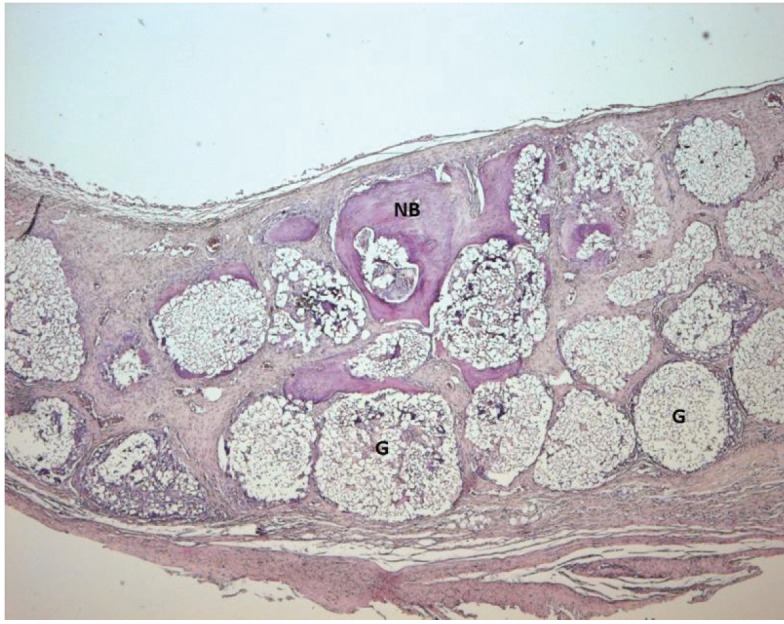
Light microscope photograph of a section from TCP group. The new bone (NB) can be seen around graft material (G) (H&E × 100).

**Figure 6:**
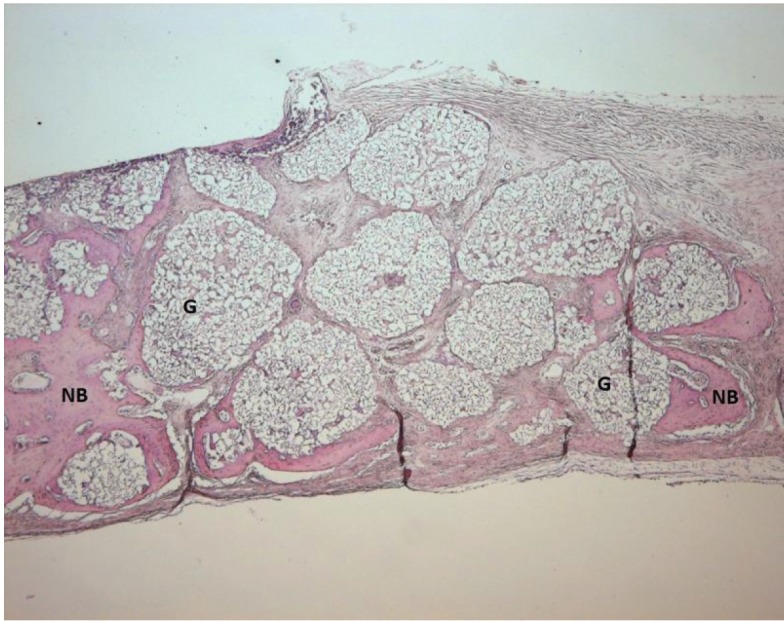
Light microscope photograph of a section from SA-PRP+TCP group. The photograph shows new bone (NB) around graft material granules (G) (H&E × 100).

**Figure 7:**
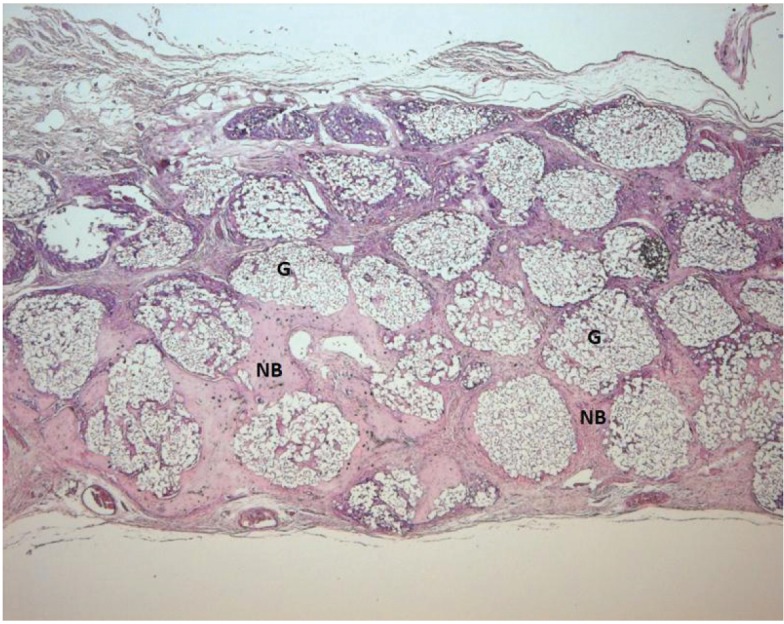
Light microscope photograph of a from DA-PRP+TCP group. The graft granules (G) are surrounded by cellular and lamellar new bone (NB) (H&E × 100).

Light microscope photographs of haematoxylin & eosin-stained sections from each group are shown in Figures 2, 3, 4, 5, 6, 7. In control group, minimal amount of new bone formation and chronic inflammatory infiltrate on fibrous connective tissue were seen (Fig. 2). In SA-PRP and DA-PRP groups, cellular new bone formation on vascularised connective tissue ground was observed (Figs. 3,4). In TCP, SA-PRP+TCP, and DA-PRP+TCP groups, fibrous connective tissue filled around granular graft material and in their neighbourhood cellular, new bone formation in close connection with granules was seen (Figs. 5, 6, 7). No foreign body reaction or osteoclast activity was detected. Lamellar bone formation in especially DA-PRP+TCP group indicated matured new bone. 

**Figure 8:**
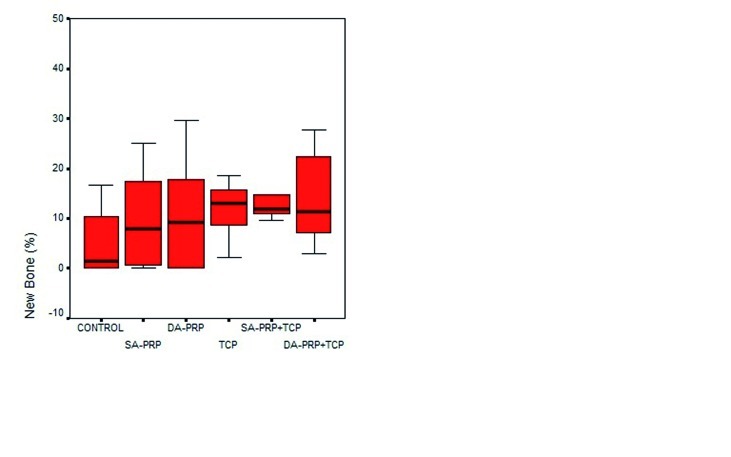
Minimum-maximum values and 50th (median), 25th and 75th percentiles of New Bone (%) for the study groups.

**Figure 9:**
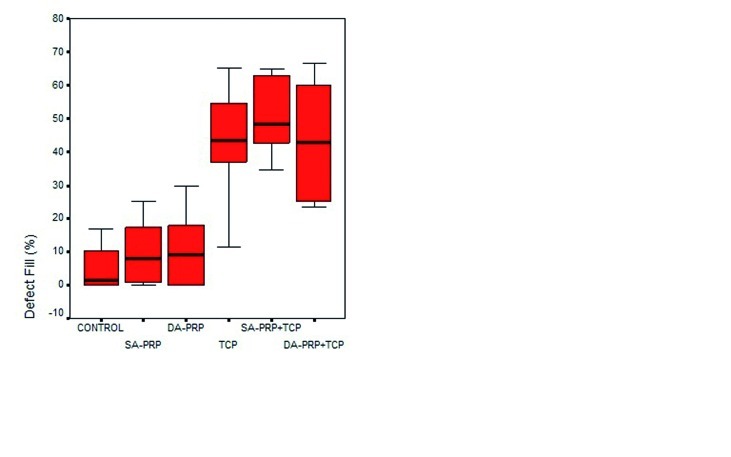
Minimum-maximum values and 50th (median), 25th and 75th percentiles of Defect fill (%) for the study groups. * The difference between Control group (p<0,001). The difference between SA-PRP group (p<0,001). The difference between DA-PRP group (p<0,001).

Histomorphometric results for NB % and DF % were summarized at (Figs. 8, 9). Minimum-maximum values as well as 50th (median), 25th and 75th percentiles are shown at (Figs. 8,9). 

The highest NB % was calculated in defects of TCP group, followed by SA-PRP+TCP, DA-PRP+TCP, DA-PRP, SA-PRP and Control groups, respectively (Fig. 8). The NB % differences among groups were not statistically significant. 

The each of the three groups treated with TCP, regardless of any PRP application addition, showed significantly more DF% than DA-PRP, SA-PRP and Control groups (p<0,001) (Fig. 9).

## Discussion

In the present study we evaluated the effect of double application of PRP on bone healing in a rabbit calvarial defect model.

For this purpose, we created two circular bone defects with 10 mm diameter on each rabbit calvaria. Previously, the critical size for rabbit calvarial bone defects was described as 15 mm in diameter (30). However, it is also known that creating multiple critical size bone defects on rabbit calvaria is not always possible due to the small size of rabbit calvaria (27). Kroese-Deutman et al. compared healing of rabbit calvarial bone defects with 6, 9 and 15 mm diameters and concluded that defect diameter did not affect on new bone percentage at the defect area (31). 

In accordance with the previous reports (32,33), we found that addition of PRP to b-TCP granules made manipulation of graft granules easier and enhanced graft stabilization along with immobilization of graft in the defect area. 

Histomorphometric data of the present study suggested that all treatment modalities were successful at the treatment of rabbit calvarial bone defect. Each resulted with more new bone formation than empty control group at the end of the one month; however no statistically significant difference was detected among groups. 

Aghaloo et al. (27) evaluated PRP application alone and compared with empty controls in a rabbit non-critical size cranial defect model at first, second and fourth months after surgery. They reported that defects treated with PRP did not showed significant advantages over controls either with histological or radiographical examination (27). Furthermore, Miloro et al. (34) stated that addition of PRP alone did not provide any statistically significant benefit to healing in an osteotomized defect of rabbit mandible at first, second and third months after surgery. On the other hand, Fontana et al. (35) examined osteogenesis around titanium implants placed at rat tibia with or without PRP and reported significantly more new bone formation at sites treated with PRP after four weeks. Mariano et al. (36) histologically analyzed the influence of PRP on bone healing in surgically created critical-size defects in the calvaria of diabetic rats at the end of a month and found that PRP significantly enhanced bone healing both qualitatively and quantitatively. The conflictive results of the different studies about treatment with PRP alone may be due to different defect models, as well as different evaluation techniques and periods. The present data showed that PRP alone did not add significant benefits to the wound healing at the end of the first month, when compared to empty controls and any other treatments evaluated. 

In our study, prosperous new bone formation and significantly higher defect fill was observed more in the groups treated with b-TCP. Yazawa et al. (10) reported considerably more new bone formation at the rabbit calvarial and mandibular intrabony defects treated with both TCP and PRP from the first day to first, fourth and eighth weeks. Kovacs et al. (1) evaluated healing at beagle dog bilateral mandibular defects treated with either TCP or PRP/TCP combination at sixth and 12th weeks. They reported that at post-op sixth week rounded TCP granules were surrounded by a fibrous encapsulations at TCP groups, where as TCP granules were surrounded by cell-rich mesencymal tissues at PRP/TCP group (1). Connective tissue islands and osteoid bridging were described as characteristics in the pores of the granules, as a major difference from group treated TCP alone (1). Intragranular budding of the richly capillarised osteogenic mesenchyma and osteoid bridges were reported to be more visible in the pores of the granules of PRP/TCP treated group at 12th week (1). When new bone formation was quantified with histomorphometric procedures, authors declared that no significant difference was detected between the groups at six weeks, whereas significantly more new bone formation was detected in PRP/TCP group compared to TCP group (1). Moreover, in a similar study model, with the histomorphometric evidences authors suggested that the newly formed bone at 12th week with PRP/TCP treated defects had the similar quality with autogenous bone (11). In the present study, no significant differences were detected between TCP, SA-PRP+TCP and DA-PRP+TCP groups at the end of the one month from the stand point of new bone formation or defect fill. When current data considered together with the results of the previous studies, we think that evaluating longer monitoring periods together with the results of first month healing may add different aspects to the evaluation of double application of PRP. Although the PRP is believed to be effective mainly at the early stages of the hard tissue healing, more evidence is needed to understand the extents of this effect and the most appropriate time of evaluation. 

Previously, some promising results of multi application of PRP via injection (15,16) or topically application (13,14) for face and neck rejuvenation, scar attenuation, treatment of chronic diabetic ulcers and degenerative lesions of articular cartilage of the knee was reported in medicine. To our knowledge, present study appears to be the first one to examine the influence of double-application of PRP in experimental bone healing model. 

In the present study, it was histomorphologically observed that newly formed bone trabeculae in double-application of PRP groups were also more mature and lamellar than both control and single-application PRP groups either with or without TCP. In particular, less osteocyts per unit tissue and less lamellar lining was detected within new bone trabeculae of control group, which both emphasized the limited maturation of bone. The histomorphological analyses revealed a remarkable increase in new bone formation with the addition of double-application of PRP. All histomorphometric analyses were carried out with an image analyse software programme which allow us to have quantitative results in addition to our histomorphological observations. Although our histomorphological findings were in favour of double-application of PRP, statistical analyses of the quantitative data did not revealed a significant increase in defect fill or new bone formation with the addition of double-application of PRP. Furthermore, in the present study the sample size was small, consisting of 8 to 10 defects in each group, which may have contributed to the lack of statistical significance between groups. 

We think that more evidences are required for the better understanding of potential of double-application of PRP at the treatment of intrabony defects. Although it is well known that animals have different regeneration capability from humans (37) and experimental studies do not give the same results as human clinical studies, still we think that current findings can form the starting point of further clinical human research in the future. Further studies conducted with longer evaluation intervals for newly formed bone and defect fill, as well as studies evaluating immunohistochemical markers of bone formation within postoperative first month are needed to clarify the additional effects of double application of PRP.
